# *LYPD3*, a New Biomarker and Therapeutic Target for Acute Myelogenous Leukemia

**DOI:** 10.3389/fgene.2022.795820

**Published:** 2022-03-11

**Authors:** Tingting Hu, Yingjie Zhang, Tianqing Yang, Qingnan He, Mingyi Zhao

**Affiliations:** ^1^ Department of Pediatrics, Third Xiangya Hospital, Central South University, Changsha, China; ^2^ College of Biology, Hunan University, Changsha, China

**Keywords:** acute myelogenous leukemia, COX, LASSO, LYPD3, poor prognosis, p53, PI3K/AKT, GSEA

## Abstract

**Background:** Acute myelogenous leukemia (AML) is nosocomial with the highest pediatric mortality rates and a relatively poor prognosis. *C4.4A*(*LYPD3*) is a tumorigenic and high-glycosylated cell surface protein that has been proven to be linked with the carcinogenic effects in solid tumors, but no hematologic tumors have been reported. We focus on exploring the molecular mechanism of *LYPD3* in the regulation of the occurrence and development of AML to provide a research basis for the screening of markers related to the treatment and prognosis.

**Methods:** Datasets on RNA Sequencing (RNA-seq) and mRNA expression profiles of 510 samples were obtained from The Cancer Genome Atlas Program/The Genotype-Tissue Expression (Tcga-gtex) on 10 March 2021, which included the information on 173 AML tumorous tissue samples and 337 normal blood samples. The differential expression, identification of prognostic genes based on the COX regression model, and LASSO regression were analyzed. In order to better verify, experiments including gene knockdown mediated by small interfering RNA (siRNA), cell proliferation assays, and Western blot were prefomed. We studied the possible associated pathways through which *LYPD3* may have an impact on the pathogenesis and prognosis of AML by gene set enrichment analysis (GSEA).

**Results:** A total of 11,490 differential expression genes (DEGs) were identified. Among them, 4,164 genes were upregulated, and 7,756 genes were downregulated. The univariate Cox regression analysis and LASSO regression analysis found that 28 genes including *LYPD3*, *DNAJC8*, and other genes were associated with overall survival (OS). After multivariate Cox analysis, a total of 10 genes were considered significantly correlated with OS in AML including *LYPD3*, which had a poor impact on AML (*p* <0.05). The experiment results also supported the above conclusion. We identified 25 pathways, including the E2F signaling pathway, *p53* signaling pathway, and *PI3K_AKT* signaling pathway, that were significantly upregulated in AML samples with high *LYPD3* expression (*p* < 0.05) by GSEA. Further, the results of the experiment suggested that *LYPD3* participates in the development of AML through the *p53* signaling pathway or/and *PI3K/AKT* signaling pathway.

**Conclusion:** This study first proved that the expression of *LYPD3* was elevated in AML, which was correlated with poor clinical characteristics and prognosis. In addition, *LYPD3* participates in the development of AML through *p53 or/and* the *PI3K/AKT* signaling pathway.

## Introduction

Acute leukemia (AL) is reported to be the 10th most common cancer all over the world with over 350,000 new cases diagnosed per year (Voelker, 2019). AL can be roughly divided into acute lymphoblastic leukemia (ALL) and acute non-lymphoblastic leukemia (ANLL), and acute myelogenous leukemia (AML) is a subtype of ANLL (Castagnola et al., 2010). AML is the deadliest form of hemal tumor worldwide, and according to the reports from the Global Burden of Disease (GBD), there were 147,000 deaths in 2015 (Mortality, 2016; Fitzmaurice et al., 2017). AML is more likely to occur in children and adolescents, making up 15–20% of AL in children and about 33% in adolescents (Creutzig et al., 2018). There is convincing evidence of a link between abnormal expression, factors of the oxidative stress, activation of various cytokines, signaling pathways, and proliferation and metastasis of tumor cells (Milkovic et al., 2014; Wee and Wang, 2017; Iqbal et al., 2019).

It has been reported that high-glycosylated cell surface proteins are overexpressed or abnormally expressed in several kinds of cancers, including breast cancer, pancreatic cancer, colon cancer, and carcinoma of the ovary. Notably, it is well documented that the abnormal expressions are related to the poor prognosis (Hollingsworth and Swanson, 2004; Vincent et al., 2008; Kitamoto et al., 2011; Gupta et al., 2012; Yang et al., 2015; van Putten and Strijbis, 2017). Therefore, what do high-glycosylated cell surface proteins have to do with cancers? According to the universally accepted views, high-glycosylated cell surface proteins promote oncogenic effects by their glycosylated extracellular domains, which might protect cancer cells under harmful conditions, and by the intracellular domain that is associated with pathways that regulate inflammation, apoptosis, and cell differentiation (van Putten and Strijbis, 2017). In addition, cancer cells appear to utilize the high-glycosylated cell surface proteins to regulate detachment and reattachment during metastasis (Maher et al., 2011). Furthermore, there is more evidence that high-glycosylated cell surface proteins are associated with cellular growth, differentiation, transformation, adhesion, invasion, and immune surveillance (Hollingsworth and Swanson, 2004; Chauhan et al., 2006; Ohtsubo and Marth, 2006; Acar et al., 2008; Maher et al., 2011). However, the molecular biomarkers which are available for early diagnosing and prognosis prediction are still required to be further studied and developed.

C4.4A (*Ly6/PLAUR domain-containing protein 3*, *LYPD3*), first reported in 1998, is a tumorigenic and high-glycosylated cell surface protein that has been proven to be linked with the carcinogenic effects in different solid tumors (Rösel et al., 1998; Hansen et al., 2004; Hansen et al., 2008; Jacobsen et al., 2012; Görtz et al., 2017; De Loma et al., 2020; Yue et al., 2020). The elevated expression of *LYPD3* is not only demonstrated to be associated with lung adenocarcinoma carcinogenesis and poor prognosis (Jacobsen et al., 2014; Cohen et al., 2017; Hu et al., 2020) but also there is evidence that *LYPD3* can lead to the initiation and development of cancers and the chemoresistance of metastatic cancers by impacting the proliferation and apoptosis of the tumor, which are involved in many important regulatory mechanisms of cancers (Lamouille et al., 2014; Fischer et al., 2015). Furthermore, the relationship between the molecule and AML remains unclear. Also, *LYPD3* has not been reported to be found in normal blood, and in our previous bioanalysis, we found that *LYPD3* expression was increased in AML, suggesting that it may be an emerging marker. *LYPD3* can be the ideal target for the therapy method and early detection of AML.

In our study, we analyzed the relationship between the expression of *LYPD3* and the clinical variables of AML according to the data obtained from the public database, and they were experimentally validated. Based on these studies, we aimed to determine the clinical value and prognostic significance of *LYPD3* in patients with AML based on our study so as to provide a theoretical basis and molecular basis for future clinical and basic research.

## Materials and Methods

### Data Source

**FIGURE 1:**
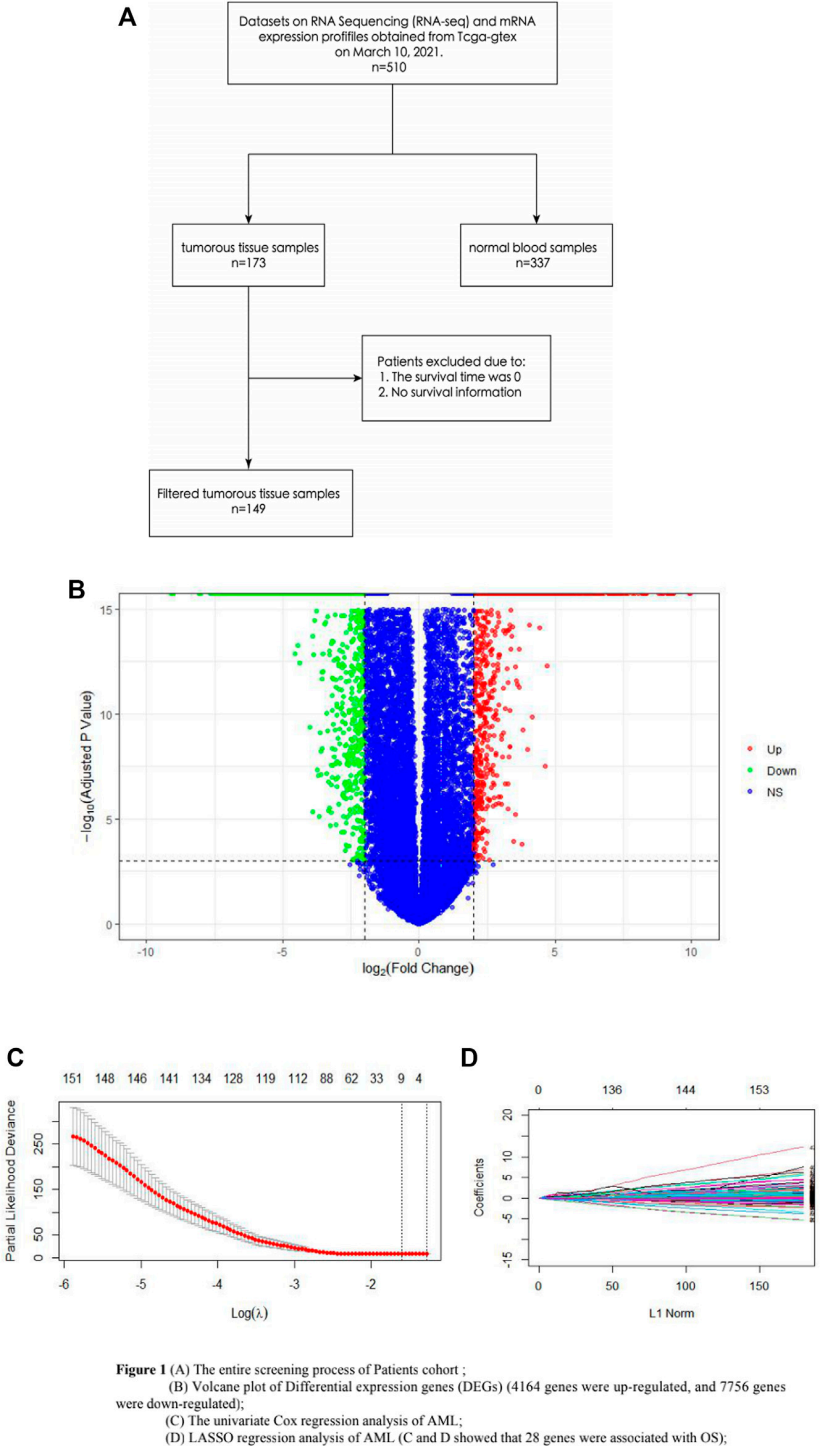
**(A)** Entire screening process of the patient cohort; **(B)** volcano plot of DEGs (4164 genes were upregulated, and 7756 genes were downregulated); **(C)** univariate Cox regression analysis of AML; and **(D)** LASSO regression analysis of AML. **(C,D)** show that 28 genes were associated with OS.

Datasets on RNA Sequencing (RNA-seq) and mRNA expression profiles of 510 samples were obtained from TCGA-GTEx on 10 March 2021, which included the information on 173 AML tumorous tissue samples and 337 normal blood samples. Among the AML tumorous tissue samples, the survival time of 12 samples was 0, and 12 samples had no survival information, which have been all removed. Finally, a total of 149 AML samples were included in this study. Also, genes with an expression value of 0, which occupied more than 60% of all the samples, were excluded. RGene expression data and phenotype data of 337 normal whole blood samples were downloaded from the GTEx (https://www.gtexportal.org/home/) database. The abovementioned databases were obtained from UCSC XENA (https://xenabrowser.net/) (Figure 1A).

### Differential Expression Analysis

The mRNA expression levels between tumor and normal samples were analyzed by using the DESeq2 package in R software. First, genes with an expression value of 0 in more than half of the samples were excluded. Differentially expressed genes were screened with the cutoff value of adjusted *p-value* < 0.05 and | log2-fold change [FC] | > 2.

### Identification of Prognostic Genes Based on the COX Regression Model and LASSO Regression Analysis

To figure out the prognostic genes of the AML samples, the related hazard ratios (HRs), 95% confidence intervals (CIs) of the HRs, and *p*-values were analyzed using univariate and multivariate Cox regression. Least absolute shrinkage and selection operator (LASSO) Cox regression analysis, which could reduce the dimensionality and select the most robust markers to predict prognosis, was used after the univariate Cox analysis to further identify the candidate genes that could be used to construct the multivariate Cox regression model. Survival analysis was conducted using the R Bioconductor. The survival package was used for univariate and multivariate Cox regression analyses, while the glmnet package was used for the LASSO regression analysis.

### Gene Enrichment Analysis

Genes in each module were subjected to Gene Ontology (GO) and Kyoto Encyclopedia of Genes Genomes (KEGG) pathway analysis to understand their biological function better. The GO enrichment analysis and KEGG signal pathway analysis of differential expression genes (DEGs) were carried out by clusterProfiler in R software, and the results were visualized. For this study, we analyzed the gene set named h.all.v6.2 symbols.gmt with the cutoff value of normal *p* < 0.05.

### Single Gene Enrichment Analysis

The Gene Set Enrichment Analysis (GSEA) software was used for single-gene enrichment analysis aiming at *LYPD3*. Based on LASSO regression and Cox regression, survival analysis was performed to screen out the prognostic genes. Finally, the receiver operating characteristic (ROC) curve was plotted, and the 1-, 3, and 5-year survival rates were calculated.

### Cell Culture

HL-60 cells (non-adherent human acute myelogenous leukemia cells), A549 cells (adherent human lung cancer cells), HCT 116 cells (adherent human colon cancer cells), EC cells (human umbilical vein endothelial cells), and CEM cells (non-adherent human lymphoma cells) were all obtained from the ScienCell (San Diego, California, United States). They were all incubated with RPMI Medium Modified medium (Cytiva, Dana Hector, United States) containing 10% fetal bovine serum (Gibco, Thermo Fisher Scientific, Waltham, MA, United States). The cells were cultured using a previously described method (Chen et al., 2018).

### *LYPD3* Gene Knockdown Mediated by Small Interfering RNA (siRNA)

We obtained *LYPD3* siRNA and scrambled negative control siRNA for transfection from Shanghai GenePharma (China). The siRNA sequences were *LYPD3* siRNA (sense: 5′- *GCU​GUA​ACU​CUG​ACC​UCC​GCA​ACA​A*-3′; antisense: 5′ *UUG​UUG​CGG​AGG​UCA​GAG​UUA​CA GC*-3′) and scrambled negative control siRNA (sense, 5′-*UUC​UCC​GAA​CGU​GUC​ACG​UTT*-3′; antisense, 5′-*ACG​UGA​CAC​GUU​CGG​AGA​ATT*-3′). HL-60 cells (0.4×10^6^ cells/well) were seeded in six-well plates and grown to 30% confluence. The HL-60 cells were divided into three groups: a control group, a blank group (transfected with negative control siRNA), and an Si-*LYPD3* group (transfected with *LYPD3* siRNA). Lip2000 (Invitrogen, China) was used to improve the transfection efficiency. The transfection mixture was replaced after 24 h with 1640 with 10% fetal bovine serum (FBS). Then, the cells were incubated for another 24 h and subjected to Western blot analysis. Also, *LYPD3* knockdown was confirmed by Western blot.

### Cell Proliferation Assays

The effect of *LYPD3* on HL-60 proliferation was assayed using the Cell Counting Kit-8 (CCK8, Beyotime, China). In summary, for the CCK8 assay, cultured HL-60 were suspended in a culture medium with 0.1% FBS and inoculated in a 96-well plate (1 × 10^4^cells/well) along with 0, 5 μM siRNA(Shanghai GenePharma, China). After 0, 24, 48, and 72 h incubation, 10 μL of CCK8 solution was added to each well. The plate was incubated for an additional 1.5 h before measuring the absorbance at a 450 nm wavelength using a microplate reader (Thermo MK3, United States).

### Western Blot

For the analysis of HO-1, Nrf2, Cas-3, Cas-1, PARP-1, akt-1, P-akt, P53, and *β*-actin at the protein level, HL-60 cells were seeded in six-well plates with a density of 7.35 × 10^5^cells per well. After 24 h, the medium was changed. Each sample was homogenized in 3 mL of lysis buffer [50 mm Tris (pH 8.0), 150 mm NaCl, and 0.5% NP40] with protease inhibitors (1 mm phenylmethylsulfonyl fluoride, 10 μg/ml aprotinin, and 10 μg/ml leupeptin), followed by incubation on ice for 30 min. After centrifugation at 15,000g for 10 min at 4°C, the protein content of the cell lysates was determined by the Bio-Rad protein assay (Hercules, CA). Twenty-five micrograms of protein per lane (unless otherwise noted) was resolved by 7.5% sodium dodecyl sulfate-polyacrylamide gel electrophoresis (SDS-PAGE) and transferred to the nitrocellulose membrane. The membranes were blocked with 1% non-fat dried milk in 50 mM Tris (pH 7.5) with 150 mM NaCl and 0.05% Tween-20 and sequentially incubated with antibodies against *LYPD3* (EPR9107, #ab1517-9, ABCAM, MA, United States), *β*-actin (SANYING, Wuhan, China), Cas-1(PROTEINTECH, United States), Cas-3(PROTEINTECH, United States), PARP-1(PROTEINTECH, United States), P-akt(PROTEINTECH, United States), akt-1(PROTEINTECH, United States), P53(PROTEINTECH, United States), and the appropriate horseradish peroxidase-conjugated, secondary anti-mouse (for ERα and *β*-actin), or anti-rabbit (for ERβ) antibodies (Amersham Biosciences, Piscataway, NJ). Blots were visualized using enhanced chemiluminescence (ECL Plus) reagents as recommended by the manufacturer (Amersham Biosciences). Densitometric analysis of band intensities was conducted using optical scanning and qualification with ImageJ. After the analysis was completed, protein expression was normalized to *β*-actin and compared to the corresponding vehicle controls.

### Statistical Analysis

All experiment data are expressed as mean ± standard deviation (SD). Graph Prism 8 software was used for statistical analysis. Mean values of the experimental groups were compared by the *t*-test and Chi-square test, and *p-value* < 0.05 was accepted as statistically significant. Western blot results were analyzed with ImageJ software. Experiments were repeated in triplicate, with similar results each time, and the figures show representative experimental results.

## Results

### Differentially Expressed Genes in the AML Sample and Normal Sample

The DEGs of 149 AML samples obtained from the TCGA database and 337 normal whole blood controls from the GTEx database were identified. To sum up, a total of 11,490 DEGs were identified. Among them, 4164 genes were upregulated and 7756 genes were downregulated (Figure 1B).

### Identification of AML-Related Genes Associated With OS

The univariate Cox regression analysis and LASSO regression analysis showed that 28 genes were associated with OS (Figures 1C,D). In order to identify the prognosis genes of AML, the multivariate Cox regression model was performed. Also, multivariate Cox analysis further narrowed these candidates into 10 genes, including *AC108479.3*, *FAM207A*, *GS1-304P7.1*, *LINC00540*, *LYPD3*, *NDST3*, *RP11-379P1.4*, *RP11-615I2.1*, *RP11-672L10.6*, *and TREML2*, which were significantly correlated with OS in AML; *AC108479.3*, *NDST3*, *RP11-379P1.4*, and *RP11-672L10.6* benefited AML, while *FAM207A*, *GS1-304P7.1*, *LINC00540*, *LYPD3*, *RP11-615I2.1*, *and TREML2* had a poor impact on AML (Supplementary Table S1). Surprisingly, *LYPD3* stands out from these 10 genes and shows a potentially negative impact on AML (*p* <0.05).

### Expression of *LYPD3* in Cancer Lines

**FIGURE 2:**
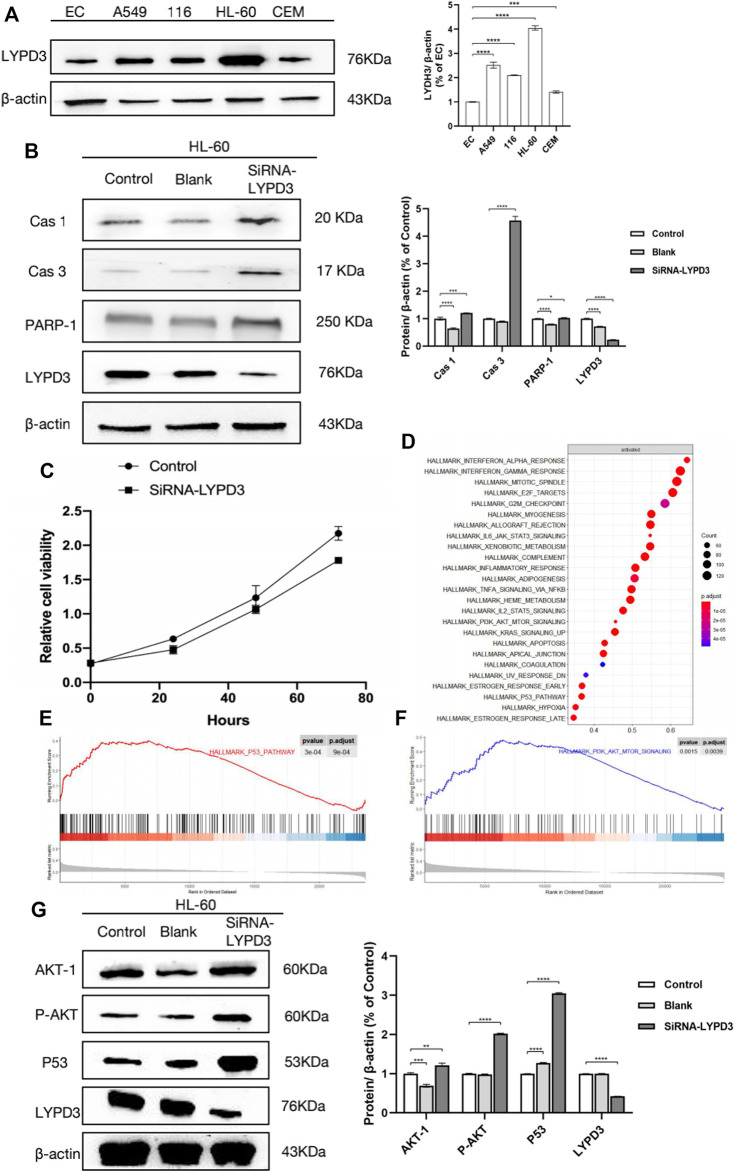
**(A)** Expression of *LYPD3* in cancer lines (the expression of *LYPD3* is the highest in HL-60 cells); **(B)** *LYPD3* gene knockdown mediated by siRNA-induced apoptosis in AML cells (HL-60 cells); **(C)** *LYPD3* gene knockdown mediated by siRNA-suppressed proliferation in AML cells (HL-60 cells); **(D)** significantly enriched pathways in AML samples with high *LYPD3* expression; **(E)** significantly enriched pathways (the P53 signaling pathways); **(F)** significantly enriched pathways (PI3K_AKT signaling pathway); **(G)** relationship between *LYPD3* and the molecules Akt and P53 (the expression of the *LYPD3* gene knockdown-mediated SiRNA group was obviously increased in p53 and PI3K_AKT signaling).

To validate the expression of the *LYPD3* protein with the *LYPD3* GPI Ab in cancer lines (see Figure 2A), a Western blot analysis was performed. This antibody gave prominent bands around 76 kDa in the A549, hct 116, and HL-60 cells, while no bands were shown in EC and CEM cells, and the results of these are consistent with the study reported by Ping Hu et al. in 2020, the research reported by Willuda J et al. in 2017, and the study reported by Wang L in 2017 (Wang et al., 2017; Willuda et al., 2017; Hu et al., 2020). As shown in Figure 2A, the color of HL-60 cell bands was darker than that of the other two bands (that is, HCT116 cell bands and A549 cell bands), indicating that the expression of *LYPD3* was the highest in the HL-60 cells. It is well documented that increased *LYPD3* expression is related to lung adenocarcinoma and colon cancer, which is the same as our experimental results mentioned above. In general, the results are expected to provide a research basis for the screening of markers related to the treatment and prognosis of AML.

### *LYPD3* Gene Knockdown Mediated by Small Interfering RNA (siRNA) Suppressed Proliferation and Induced Apoptosis in AML Cells (HL-60 Cells)

To investigate the function of *LYPD3*, proliferation and apoptosis assays were carried out in HL-60 transfected with siRNA-*LYPD3*. As shown in Figure 2C, *LYPD3* expression was significantly suppressed in HL-60 cells by siRNA-*LYPD3*. It is significant because it has to do with one of the mechanisms of leukemia, abnormal cell proliferation, which might provide a molecular basis for future clinical and basic research. After that, one is that the proliferation of HL-60 cells was evaluated more distinctly compared with the HL-60 cells by siRNA-*LYPD3*; that is to say, *LYPD3* gene knockdown mediated by siRNA-*LYPD3* suppressed proliferation. Furthermore, the expression of apoptotic markers including *CAS-1*, *CAS-3*, and *PARP-1* was significantly upregulated, indicating that obvious inducing of cell apoptosis occurred in HL-60 cells (Figure 2B). This result demonstrated that knocking down *LYPD3* induced multiple cell death modes including apoptosis, pyroptosis, and parthanatos, which suppressed the growth of HL-60 cells.

### Pathway Analysis of the Effect of *LYPD3* on AML

According to the above results, we have definitely confirmed that *LYPD3* should play an important role in AML. Thus, we further explored the possible related pathways through which *LYPD3* affected the pathogenesis and prognosis of AML. As shown in Figure 2D, the significantly enriched pathways in AML samples with high *LYPD3* expression were analyzed by GSEA. We identified 25 pathways that were significantly upregulated in AML samples with high *LYPD3* expression (Figure 2D, normal *p* < 0.05), including the E2F signaling pathway, the p53 signaling pathway, the *PI3K_AKT* signaling pathway, and so forth. Among them, the two most significantly enriched pathways were the *P53* signaling pathway and the *PI3K_AKT* signaling pathway (Figures 2E,F). In conclusion, we thought that it was doubtful that *LYPD3* might affect the clinical features of AML by regulating the *P53* and/or *PI3K_AKT* signaling pathway. To further explore our conjecture, Western blot was performed (Figure 2G). As shown, the expression of *LYPD3* gene knockdown mediated by the siRNA group was obviously increased in *p53* and *PI3K_AKT* signaling, which was consistent with our predictions. However, their specific mechanisms and the upstream and downstream molecules that regulate them need to be further studied.

## Discussion

*LYPD3*, “C4.4A”, a membrane protein, partially anchored to the cell surface by glycosylphosphatidylinositol (GPI), which showed predicted structural homology to other members of the *Ly6/uPAR (LU)* protein family (Hansen et al., 2004; Korkmaz et al., 2008; Fujihara et al., 2013; Gårdsvoll et al., 2013). The genes that encode these proteins are clustered in a tiny region on chromosome 19q13. After post-translation processing, *LYPD3* was composed of 278 amino acids distributed in a C-terminal region rich in serine and two N-terminal Lu domains of threonine (Jacobsen et al., 2014). In addition, *LYPD3* was reported to be normally mostly expressed in meningioma tissues according to the study by Mette C in 2011 (Kriegbaum et al., 2011). Furthermore, *LYPD3* has been demonstrated to be highly expressed in several human malignancies, such as breast cancer, colorectal cancer, esophageal cancer, renal cell carcinomas, and so forth (Fletcher et al., 2003; Hansen et al., 2004; Hansen et al., 2008; Miyake et al., 2015; Cohen et al., 2017; Hu et al., 2020; Monteiro et al., 2020). It was found that tumor cell expression of *LYPD3* correlates with poor prognosis in non-small cell lung cancer (NSCLC), esophageal cancer, and renal cell carcinomas. Thus, the association between *LYPD3* and cancer development is receiving increasing scientific attention and is well worth investigating. Also, studies have found that *LYPD3* is consistently associated with tumor progression and wound healing (Jacobsen and Ploug, 2008). It is also well documented that *LYPD3* can specifically be involved in tumor cell invasion through its interaction with the extracellular matrix (Paret et al., 2007). However, the role of *LYPD3* expression in the occurrence and development of AML remains unclear.

In our study, we found that a total of 11,490 DEGs were identified with 4,164 genes upregulated and 7,756 genes downregulated. Also, in order to identify the prognosis genes of AML, the multivariate Cox regression model was performed. After multivariate Cox analysis, a total of 10 genes including *LYPD3* were significantly correlated with OS in AML, and the results of univariate Cox regression analysis and LASSO regression analysis also indicated that *LYPD3* is associated with AML and poor prognosis (*p* = 0,01), which supported the theory that the expression of *LYPD3* would be closely correlated with the development of AML and might function as an oncogene for AML.

To validate the expression of the *LYPD3* protein with *LYPD3* GPI Ab in cancer lines (see Figure 2A), a Western blot analysis was performed, and the results also support the above-mentioned thought. Then, we further explored the possible related pathways through which *LYPD3* affected the pathogenesis and prognosis of AML. GSEA demonstrated that the *P53* signaling pathway and/or *PI3K_AKT* were significantly enriched in the high-*LYPD3* expression group. The incidence of AML is generally believed that the reasons for the proliferation and apoptosis of leukemia cells were inhibited (Heinrich, 2004; Kornblau et al., 2010; Shih et al., 2013; Elgarten and Aplenc, 2020). In addition, since there is no evidence that *LYPD3* plays a role in proliferative activity or resistance to apoptosis and the two above-mentioned characteristics are thought to be associated with AML pathogenesis according to the above universally recognized views, the most likely explanation may be that *LYPD3* acts as a coactivator. Moreover, it has been reported that a train of AML has intact, unaltered *P53* alleles (Ley et al., 2013; Papaemmanuil et al., 2016). Furthermore, the conundrum of infrequent *P53* mutations in AML is emphasized by the evidence that inactivation of P53 potently promotes AML (Barbosa et al., 2019). Indeed, the study also found that *P53* is one of the most powerful independent indicators of poor outcomes in AML. Thus, we proved that *LYPD3* can be involved in regulating the occurrence, invasion, and metastasis of AML. As shown in Figure 2B, the expression of apoptotic markers including *CAS-1*, *CAS-3*, *and PARP-1* was significantly upregulated, which indicated that obvious inducing of cell apoptosis occurred in HL-60 cells. The view that was widely approved was that apoptosis, the most widely studied cell death program, which can be retained as a capacity to undergo, as will be discussed as follows, contributes to both carcinogenesis and anticancer processes (Gregory and Paterson, 2018). Studies have shown that in cancer, separation from neighbors or the substrate triggers a type of spontaneous apoptotic suicide called nest-loss apoptosis. In part, nest-loss apoptosis occurs because cells are deprived of essential integrins and cadherin-mediated survival signals. However, recent studies have shown that interference with the intracellular cytoskeleton caused by detachment can directly trigger apoptosis through the release of pro-apoptotic *BH3* proteins (Evan and Vousden, 2001). In addition, *Akt* mutations are activated in the apoptotic survival signaling pathway in tumors (Russo et al., 2020). *Akt* is a serine/threonine kinase that induces strong survival signaling that is related to the loss of the inhibitor of *Akt* function *PTEN*, which is consistent with the conclusion that the expression of Akt is increased, as shown in Figure 2G. In summary, the relationship between *LYPD3*, apoptosis, and leukemia remains complex and unclear, and the specific mechanisms and the upstream and downstream molecules that regulate them need to be further studied.

## Conclusion

For the first time, we identified that *LYPD3* may promote AML progress through the *PI3K/AKT* and *p53* pathway, which provided a brand new potential biomarker and target for the clinical test and therapy of AML.

## Data Availability

The datasets presented in this study can be found in online repositories. The names of the repository/repositories and accession number(s) can be found in the article/Supplementary Material.
