# Supplementary material for: LYPD3, a New Biomarker and Therapeutic Target for Acute Myelogenous Leukemia
Source: Front Genet. 2022 Mar 11;13:795820. doi: 10.3389/fgene.2022.795820 (PMC8963240; doi:10.3389/fgene.2022.795820)
Supplement: Supplementary file 1 [file DataSheet1.PDF]

Genes	Hazard Ratio	95%CI	P-Value
GS1-304P7.1	2.32	1.14-4.73	0.021
LYPD3	1.86	1.16-2.98	0.01
RP11-615I2.1	1.63	1.12-2.37	0.011
LINC00540	1.53	1.01-2.34	0.047
RN7SL846P	1.37	0.94-1.98	0.098
RP11-385F7.1	1.14	0.79-1.65	0.474
FAM207A	1.07	1-1.15	0.042
TREML2	1.06	1-1.12	0.037
STIM2	1.03	0.99-1.08	0.144
CREB3	1.03	0.91-1.18	0.614
RP11-97O12.5	1.02	0.89-1.17	0.772
DNAJC8	1.01	0.99-1.04	0.199
CBR1	1.01	0.98-1.04	0.509
SPINK2	1	1.0-1.0	0.885
MYB	1	0.99-1	0.386
LSP1	1	1.0-1.0	0.952
CLCN5	1	0.88-1.13	0.951
RP11-584P21.4	0.99	0.81-1.2	0.9
ZNF511	0.99	0.94-1.04	0.586
LTK	0.99	0.98-1.01	0.356
SLC24A3	0.96	0.9-1.03	0.255
CTD-2260A17.1	0.86	0.72-1.02	0.078
NDST3	0.85	0.74-0.98	0.029
AC108479.3	0.77	0.65-0.92	0.004
RP11-263C24.1	0.73	0.5-1.07	0.107
ACSS3	0.58	0.32-1.06	0.078
RP11-672L10.6	0.3	0.14-0.65	0.002
RP11-379P1.4	0.25	0.1-0.61	0.002

Table 1 Multivariate Cox Regression analysis in OS

After multivariate Cox analysis, a total of ten genes were significantly correlated with OS in AML

Abbreviation: OS, overall survival; CI, confidence interval
