# Supplementary figures and images for: LYPD3, a New Biomarker and Therapeutic Target for Acute Myelogenous Leukemia
Source: Front Genet. 2022 Mar 11;13:795820. doi: 10.3389/fgene.2022.795820 (PMC8963240; doi:10.3389/fgene.2022.795820)

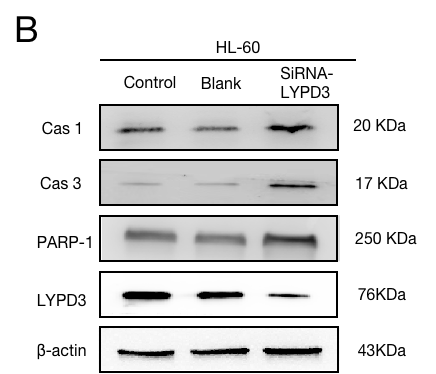

Supplement: Supplementary file 2 [file DataSheet2.ZIP › FIGURES/figure 2b.png]

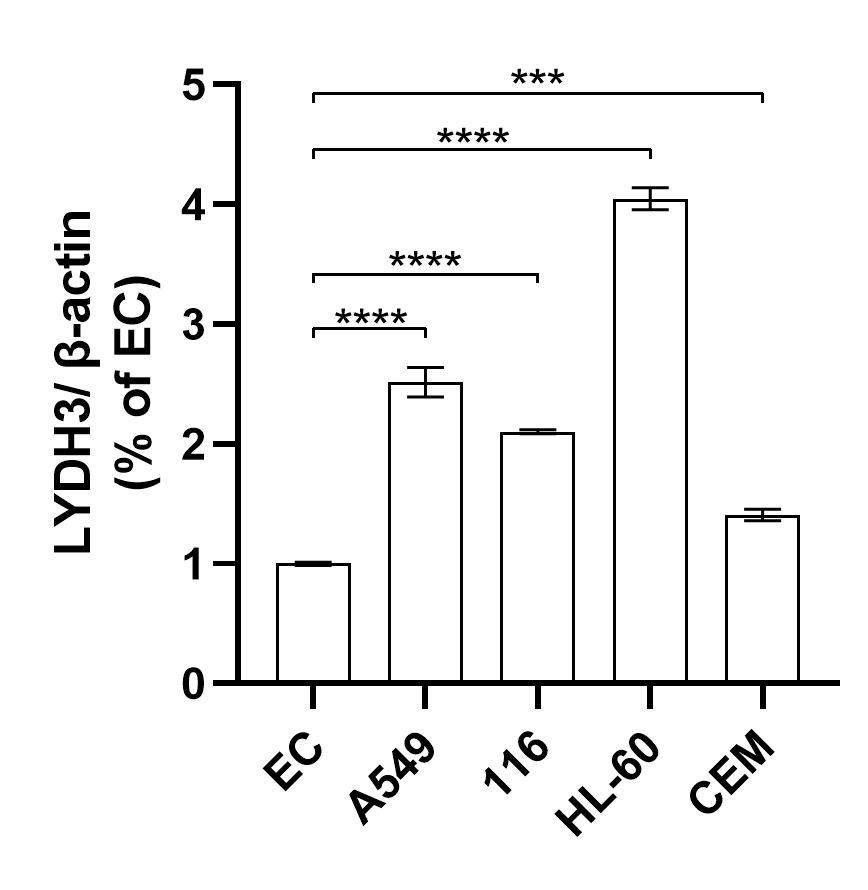

Supplement: Supplementary file 2 [file DataSheet2.ZIP › FIGURES/f2A 1.tif]

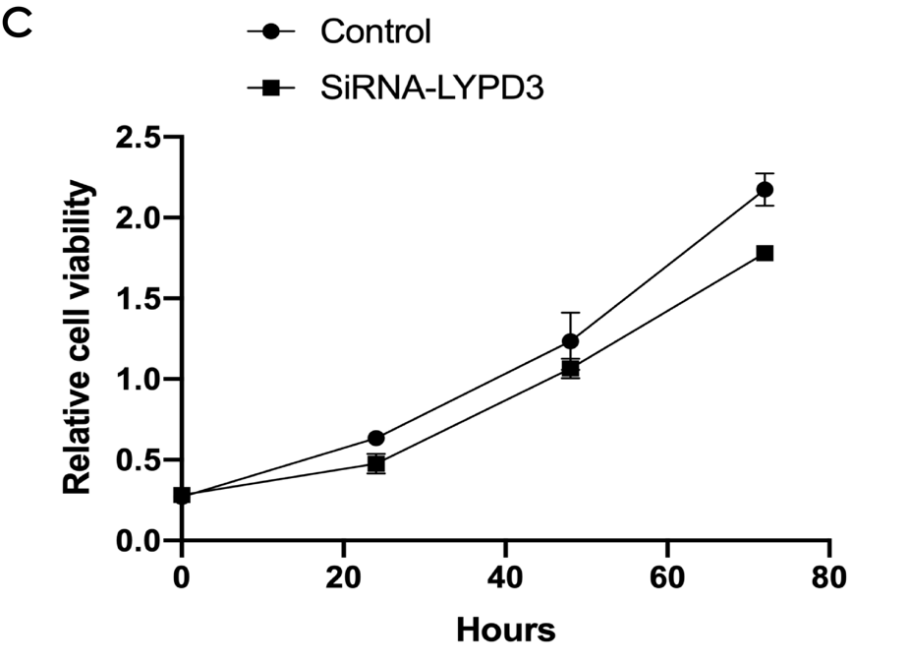

Supplement: Supplementary file 2 [file DataSheet2.ZIP › FIGURES/figure 2c.png]

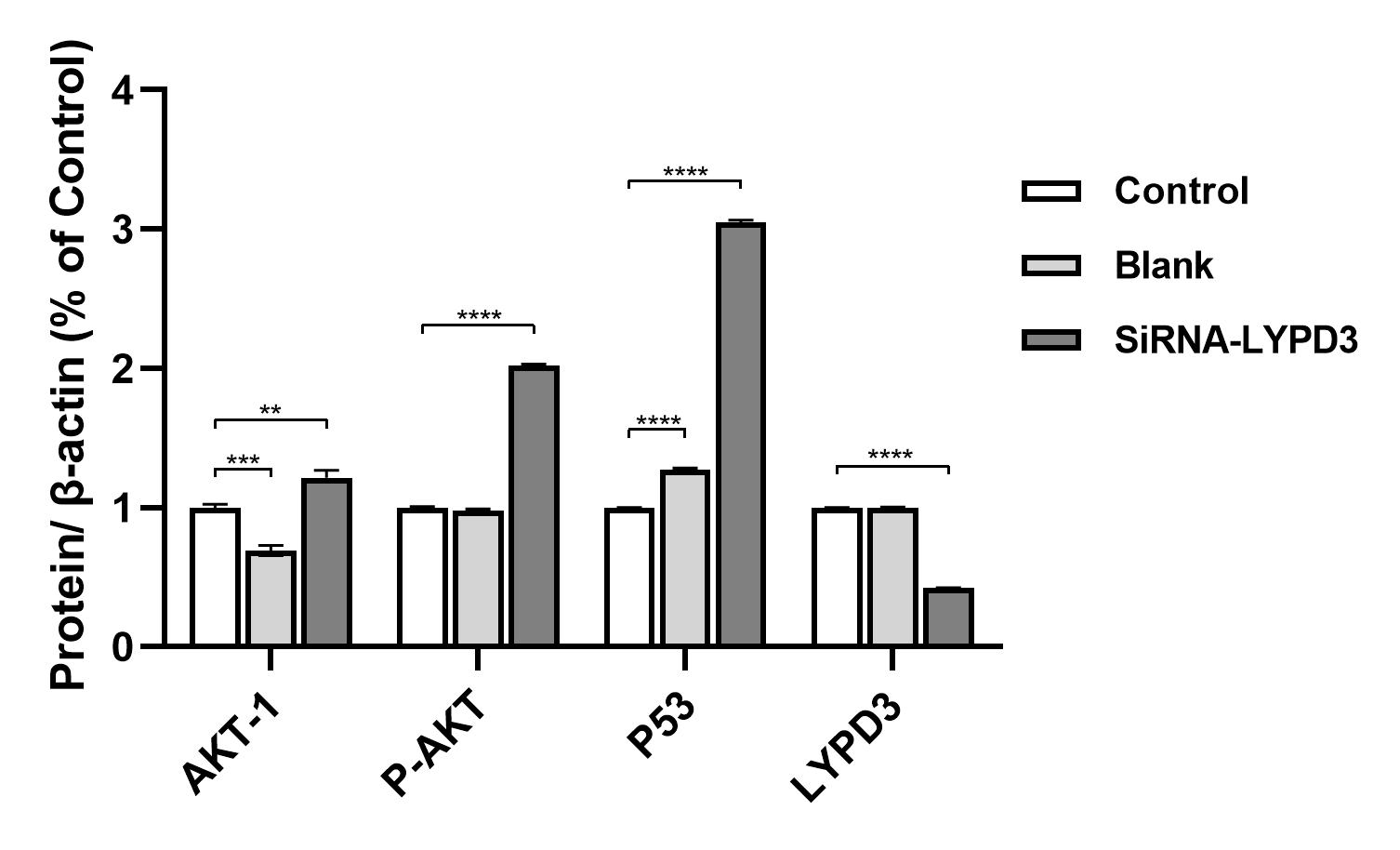

Supplement: Supplementary file 2 [file DataSheet2.ZIP › FIGURES/f3.tif]

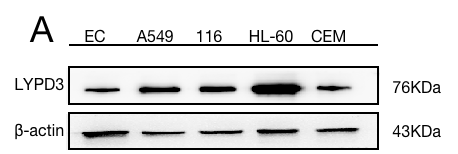

Supplement: Supplementary file 2 [file DataSheet2.ZIP › FIGURES/Figure 2a.png]

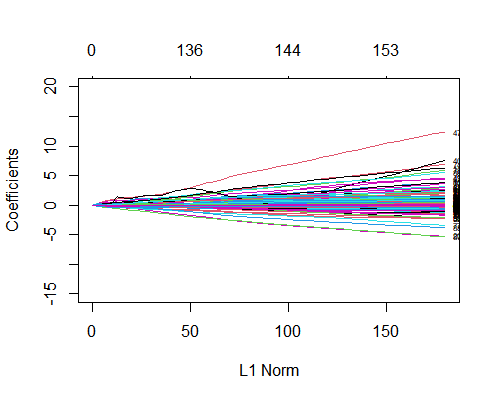

Supplement: Supplementary file 2 [file DataSheet2.ZIP › FIGURES/figure 1d.tiff]

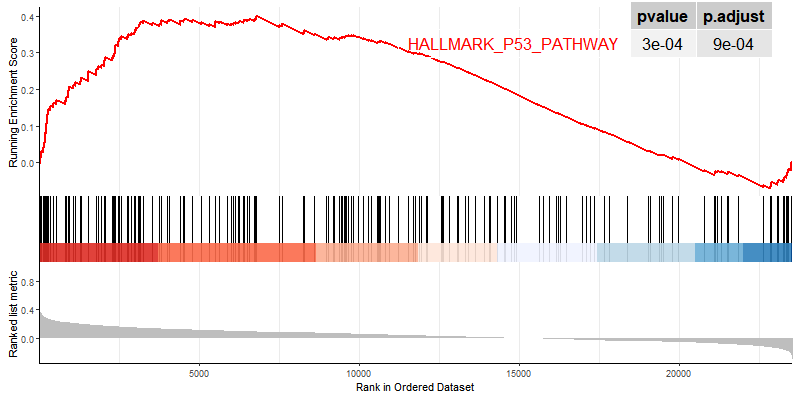

Supplement: Supplementary file 2 [file DataSheet2.ZIP › FIGURES/figure 2e.png]

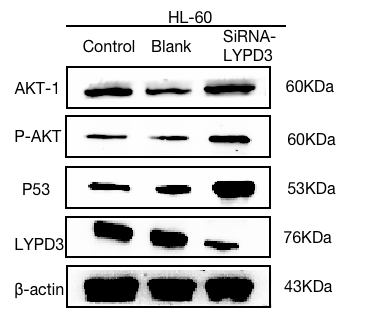

Supplement: Supplementary file 2 [file DataSheet2.ZIP › FIGURES/figure 2g.png]

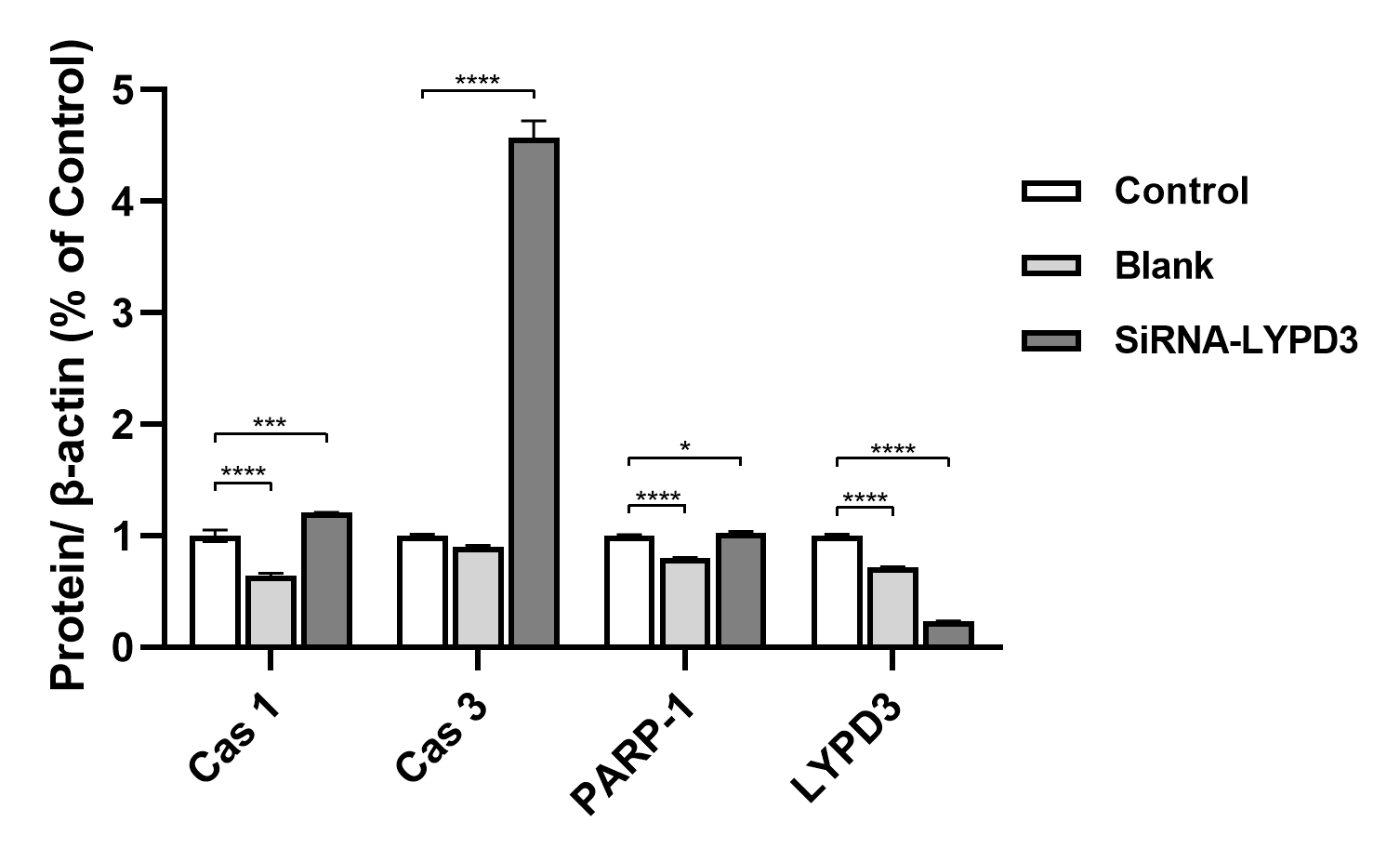

Supplement: Supplementary file 2 [file DataSheet2.ZIP › FIGURES/f2B 1.tif]

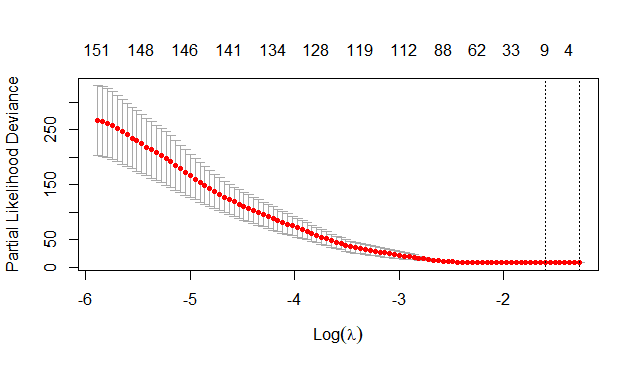

Supplement: Supplementary file 2 [file DataSheet2.ZIP › FIGURES/figure 1c.tiff]

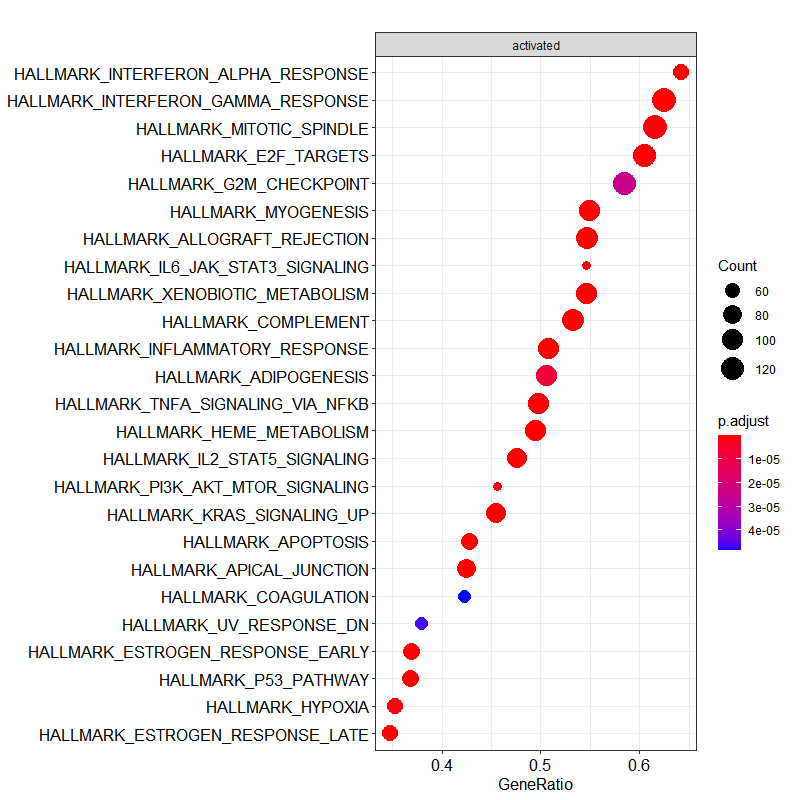

Supplement: Supplementary file 2 [file DataSheet2.ZIP › FIGURES/figure 2d.tiff]

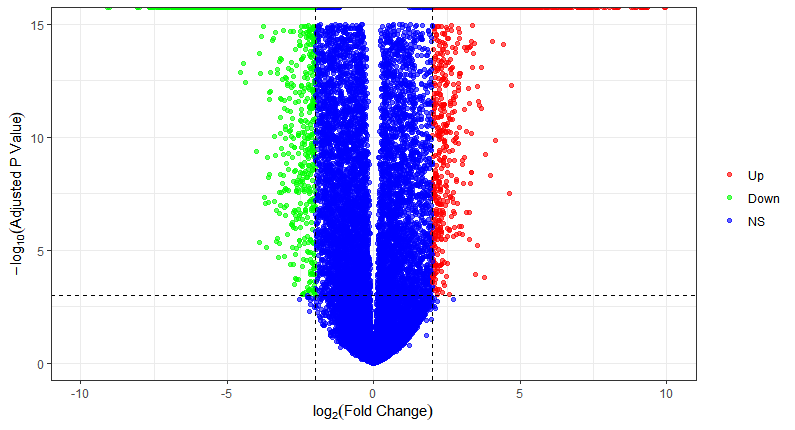

Supplement: Supplementary file 2 [file DataSheet2.ZIP › FIGURES/figure 1b.tiff]

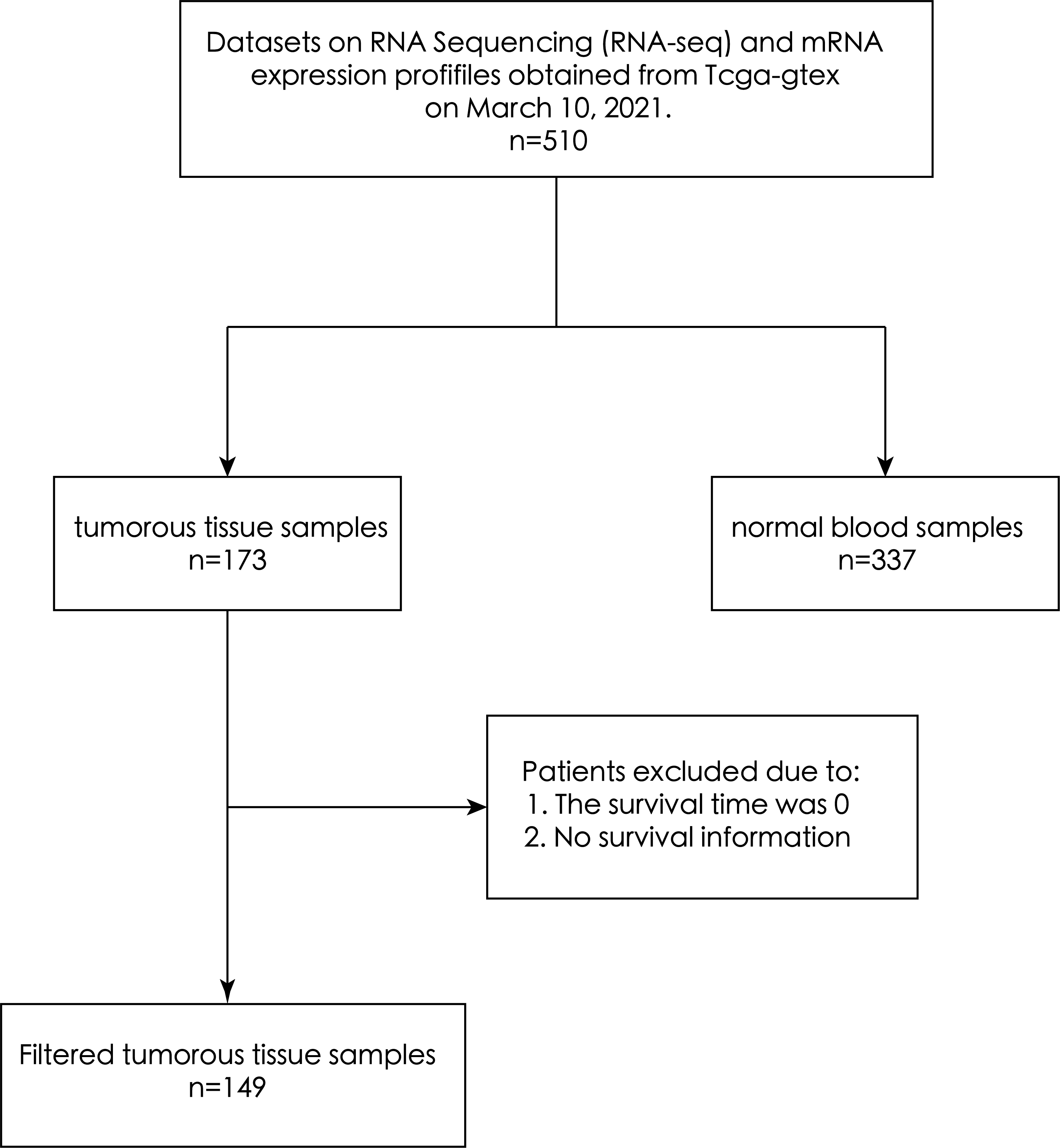

Supplement: Supplementary file 2 [file DataSheet2.ZIP › FIGURES/figure 1a.tif]

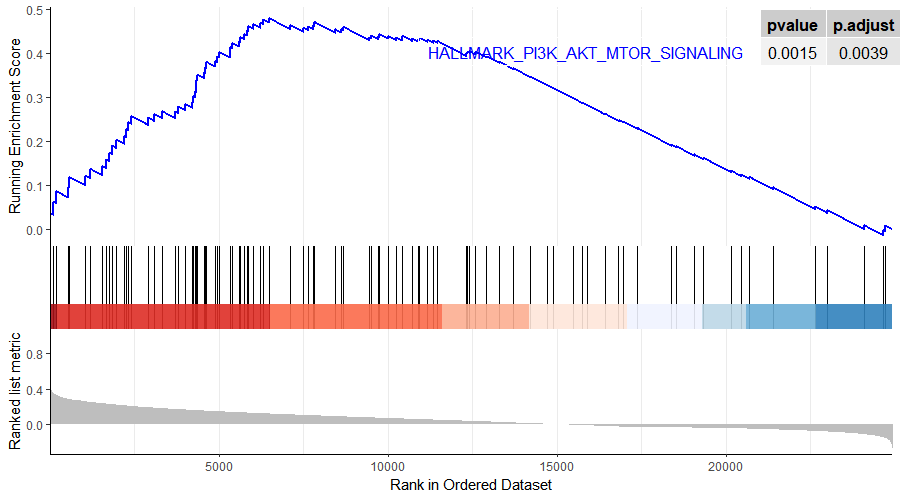

Supplement: Supplementary file 2 [file DataSheet2.ZIP › FIGURES/figure 2f.tiff]
